# Deficiency in mouse Y chromosome long arm gene complement is associated with sperm DNA damage

**DOI:** 10.1186/gb-2010-11-6-r66

**Published:** 2010-06-23

**Authors:** Yasuhiro Yamauchi, Jonathan M Riel, Zoia Stoytcheva, Paul S Burgoyne, Monika A Ward

**Affiliations:** 1 Institute for Biogenesis Research, John A Burns School of Medicine, University of Hawaii, 1960 East-West Rd, Honolulu, HI 96822, USA; 2 Division of Developmental Genetics, MRC National Institute for Medical Research, Mill Hill, London NW7 1AA, UK

## Abstract

**Background:**

Mice with severe non-PAR Y chromosome long arm (NPYq) deficiencies are infertile *in vivo *and *in vitro*. We have previously shown that sperm from these males, although having grossly malformed heads, were able to fertilize oocytes via intracytoplasmic sperm injection (ICSI) and yield live offspring. However, in continuing ICSI trials we noted a reduced efficiency when cryopreserved sperm were used and with epididymal sperm as compared to testicular sperm. In the present study we tested if NPYq deficiency is associated with sperm DNA damage - a known cause of poor ICSI success.

**Results:**

We observed that epididymal sperm from mice with severe NPYq deficiency (that is, deletion of nine-tenths or the entire NPYq gene complement) are impaired in oocyte activation ability following ICSI and there is an increased incidence of oocyte arrest and paternal chromosome breaks. Comet assays revealed increased DNA damage in both epididymal and testicular sperm from these mice, with epididymal sperm more severely affected. In all mice the level of DNA damage was increased by freezing. Epididymal sperm from mice with severe NPYq deficiencies also suffered from impaired membrane integrity and abnormal chromatin condensation and suboptimal chromatin protamination. It is therefore likely that the increased DNA damage associated with NPYq deficiency is a consequence of disturbed chromatin remodeling.

**Conclusions:**

This study provides the first evidence of DNA damage in sperm from mice with NPYq deficiencies and indicates that NPYq-encoded gene/s may play a role in processes regulating chromatin remodeling and thus in maintaining DNA integrity in sperm.

## Background

The DNA of the male specific region of the mouse Y chromosome long arm (NPYq) is highly repetitive and includes multiple copies of at least five distinct genes: *Ssty1*, *Ssty2*, *Sly*, *Asty*, and *Orly *[1,2] (J Alfoldi and DC Page, personal communication). These genes are exclusively expressed in spermatids during the final stages of spermatogenesis [1-3]. NPYq deficiency leads to teratozoospermia, subfertility with progeny sex ratio skewed towards females, or to complete infertility [4-8]. We have previously shown that infertility of mice with severe NPYq deficiencies can be overcome with intracytoplasmic sperm injection (ICSI) [8,9]; however, the overall efficiency of ICSI was unsatisfactory. This was particularly marked in further ICSI trials with frozen epididymal sperm from males lacking NPYq; despite using artificial oocyte activation, a total of 287 oocytes injected and 101 embryos transferred into 7 surrogates yielded only 1 pregnancy and 1 viable offspring (Table 1). Poor ICSI success can be due to sperm DNA damage, which is often associated with disturbed chromatin packaging [10-12]. Here, we demonstrate that severe NPYq-deficiency results in a high incidence of DNA damage in epididymal sperm, increased sperm damage due to freezing, impaired membrane integrity, poor chromatin condensation and suboptimal sperm chromatin protamination.

**Table 1:** Intracytoplasmic sperm injection with cryopreserved epididymal sperm from NPYq-^2 ^males

Experiment	Number of oocytes injected	Number of oocytes survived (%)^a^	Number of oocytes activated (%)^b, c^	Number of oocytes cleaved (%)^b^	Number of two-cell embryos transferred (number of surrogates)	Number of pregnant surrogates	Number of fetuses
1	64	34 (53)	26 (76)	17 (50)	17 (1)	0	0
2	88	65 (74)	57 (72)	26 (40)	26 (2)	0	0
3	59	44 (75)	41 (93)	25 (57)	25 (2)	1	1
4	76	47 (62)	38 (81)	33 (70)	33 (2)	0	0
1-4	287	190 (66)	162 (85)	101 (53)	101 (7)	1	1

Percentage was calculated from: ^a^oocytes injected; ^b^oocytes survived. ^c^Oocytes were chemically activated with SrCl_2_; the oocytes were considered activated when they extruded second polar body and had two well-developed pronuclei.

## Results

### Epididymal sperm from males with NPYq deficiencies are less efficient in oocyte activation after ICSI than testicular sperm

To test for sperm origin or freezing effects on ICSI outcome, injections were carried out with fresh or frozen epididymal sperm, and fresh or frozen testicular sperm. Two males were sampled for each NPY-deficient and the matched control genotypes (see Materials and methods for mouse genotype details).

**Figure 1:**
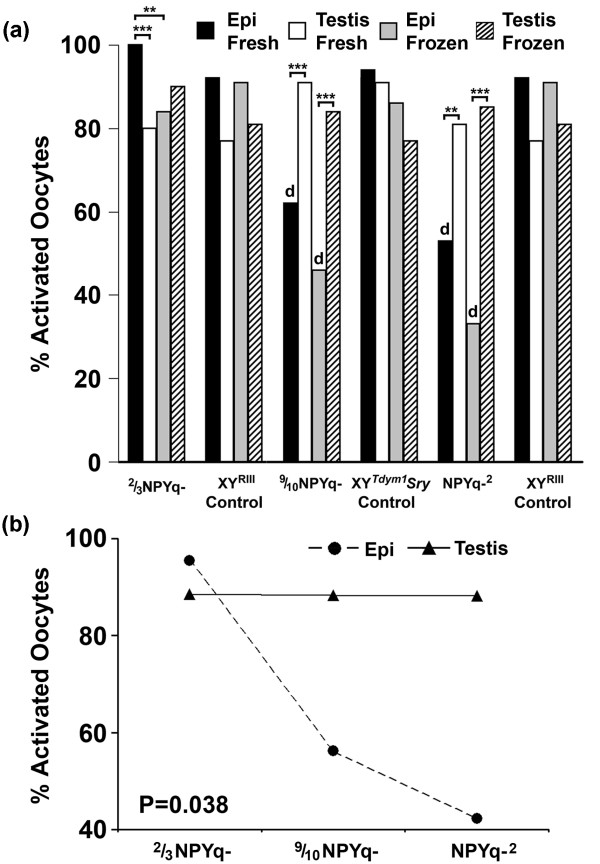
**Oocyte activation after ICSI with sperm from mice with NPYq deficiency**. (**a**) Epididymal sperm from males with severe NPYq deficiency (9/10NPYq- and NPYq-^2^) but not from males with moderate NPYq gene loss (2/3NPYq-) were less able to activate oocytes than epididymal sperm from their appropriate controls, as revealed by Fisher's exact test (d, *P *< 0.0001 versus matching sperm type in control). Epididymal sperm from males with severe NPYq deficiency (9/10NPYq- and NPYq-^2^) but not from males with moderate NPYq gene loss (2/3NPYq-) were less able to activate oocytes than testicular sperm, as revealed by Mantel-Haenszel chi square analysis (**P < 0.01, ***P < 0.001). (**b**) Genotype/source interaction revealed by ANOVA, showing that NPYq deficiency preferentially impairs oocyte activation with epididymal sperm (*P *= 0.038). Two males were used per genotype; four sperm groups (epididymal, frozen epididymal, testicular and frozen testicular) were examined per male; approximately 25 (24.88 ± 8.03) oocytes were scored per sperm group per male.

The initial analysis was carried out using the pooled data for the two males of each genotype; the numbers of activated and non-activated oocytes were compared between the NPYq-deficient genotypes and their controls using Fisher's exact test (Figure 1a). For the NPYq-^2 ^versus XY^RIII ^comparison this revealed that fresh epididymal sperm and frozen epididymal sperm from NPYq-^2 ^males were less efficient in oocyte activation than those from XY^RIII ^controls; this also proved to be the case for 9/10NPYq- (*P *= 0.0001 in all four cases). In 2/3NPYq- neither the frozen nor the fresh epididymal sperm were affected. The degree of impairment of epididymal sperm agrees well with the ranking of the genotypes with respect to the severity of the sperm head abnormalities: NPYq-^2 ^> 9/10NPYq- > 2/3NPYq [7].

A caveat to this initial analysis is that there were indications of significant inter-male variability, particularly with respect to the two NPYq-^2 ^males. We therefore carried out 'within genotype' comparisons of epididymal and testicular sperm, both fresh and frozen, keeping the individual male data separate, and testing for significant differences using Mantel-Haenszel chi square analysis, which takes account of male to male variation. The control genotypes XY^RIII ^and XY^*Tdym1*^*Sry *did not show any significant differences between epididymal and testicular sperm, whether fresh or frozen, in their ability to activate oocytes. As would be expected from the NPYq-deficient versus control comparisons, there was a significant reduction in the oocyte activation efficiency with fresh epididymal sperm as compared to fresh testicular sperm, and with frozen epididymal sperm relative to frozen testicular sperm in NPYq-^2 ^and 9/10NPYq- (Figure 1a). In contrast, the fresh epididymal sperm from 2/3NPYq- gave a significantly higher level of activation than fresh testicular sperm or frozen epididymal sperm.

The above analyses point to NPYq deficiency being a cause of impaired epididymal sperm function, with these effects being proportional to the extent of NPYq gene loss. In the light of this we decided to carry out a single analysis of all the NPYq-deficient male data by ANOVA, with genotype, sperm source (testis or epididymis) and sperm status (fresh or frozen) as factors; an identical analysis was carried out on the two control genotypes for comparison. For these ANOVAs, oocyte activation percentages for individual males were transformed into angles. No significant differences for the three factors were detected among the controls. In contrast, the analysis of the NPYq-deficient male data revealed significant effects of genotype (progressively reduced activation with increasing NPYq-deficiency; *P *= 0.036), sperm source (less activation with epididymal sperm than testicular sperm; *P *= 0.020), and a genotype/source interaction (epididymal sperm more affected by NPYq-deficiency than testicular sperm; *P *= 0.038; Figure 1b). Thus, these ANOVA analyses confirm the conclusions from the prior analyses.

We conclude that there is a reduction in oocyte activation that increases with the extent of NPYq deficiency, and this effect of NPYq deficiency is largely confined to epididymal sperm.

### NPYq deficiency is associated with sperm DNA damage

Poor activation rates can be circumvented by artificial activation but we have found that even with artificial activation ICSI success rates remained very low with frozen epididymal sperm from NPYq-^2 ^males (Table 1), so we suspected that DNA damage may be an important additional factor. We therefore performed comet assays on epididymal and testicular sperm to directly test for DNA damage.

Testicular sperm samples for comet assay were prepared in the same manner as for ICSI so they included other testicular cell types that are not present in the epididymal sperm samples. To test if the presence of these other cell types affects comet assay results, experiments were performed in which a portion of both epididymal and fresh testicular cell suspensions were sonicated under conditions that eliminate these sonication-sensitive cells; comet assays were then performed on non-sonicated and matched sonicated samples, both before and after freezing. Two males of each of the genotypes XY^RIII^, XY^*Tdym1*^*Sry*, and 2/3NPYq- were analyzed; 100 sperm comet tail lengths were measured from each male. The comet tail length data were analyzed by ANOVA with sonication status (sonicated or non-sonicated), genotype, sperm source (testis or epididymis) and sperm status (fresh or frozen) as factors. There were no significant effects of sonication so the fuller analysis including all the NPYq-deficient and control genotypes was therefore carried out without sonication.

**Figure 2:**
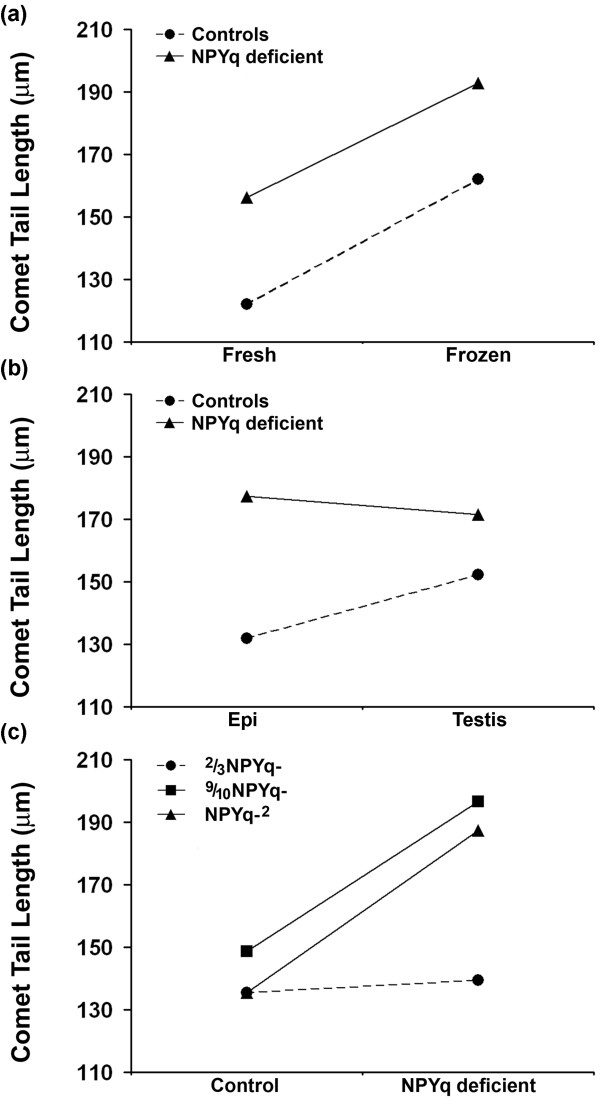
**Tail length analysis in comet assay with sperm from mice with NPYq deficiencies - ANOVA analysis**. **(a) **An increase in comet tail length due to sperm freezing in controls (*P *= 0.000001) and NPYq deficient mice (*P *= 0.0084). **(b) **An increase in comet tail length with testicular as compared to epididymal sperm in controls (*P *= 0.001) but not in NPYq deficient mice. **(c) **Comparison of NPYq deficient genotypes with their respective controls showing the significant increase in comet tail length in 9/10NPYq- (*P *= 0.0093) and NPYq-^2 ^(*P *= 0.0036). Two males were used per genotype; four sperm groups (epididymal, frozen epididymal, testicular and frozen testicular) were examined per male; 100 sperm were scored per group per male.

We first analyzed the data for the two types of control males and this showed that sperm freezing significantly (*P *= 0.000001) increased comet tail length (Figure 2a), and that testicular sperm had longer sperm comet tails than epididymal sperm (*P *= 0.001; Figure 2b). However, there was also a significant (*P *= 0.013) effect of genotype in that frozen sperm from XY^*Tdym1*^*Sry *males had approximately 25% longer comet tails than those from XY^RIII^ (a similar increased sensitivity to freezing was also apparent with sperm from 9/10NPYq-, which carry a deleted form of the Y^*Tdym1*^. We do not yet know the underlying basis for this increased sensitivity to sperm freezing in these two genotypes).

We then compared each NPYq-deficient genotype with its matched control. There were no significant differences between 2/3NPYq- and the XY^RIII ^control, but the two remaining NPYq-deficient genotypes had significantly increased sperm comet tail lengths relative to their controls (Figure 2c). Analysis of the three NPYq-deficient genotypes in a single ANOVA showed (as in controls) a significant increase of comet tail length in response to freezing (*P *= 0.0084; Figure 2a); in contrast to controls there was no significant increase in comet tail length in testicular sperm as compared to epididymal sperm (Figure 2b). Indeed, when the effect of sperm source was compared in analyses within each NPYq-deficient genotype, comet tails were significantly longer in epididymal sperm as compared to testicular sperm from NPYq-^2 ^(*P *= 0.0034), and this resulted in a highly significant genotype/sperm source interaction (*P *= 0.0004) when NPYq-^2 ^was compared with its matched XY^RIII ^control.

**Figure 3:**
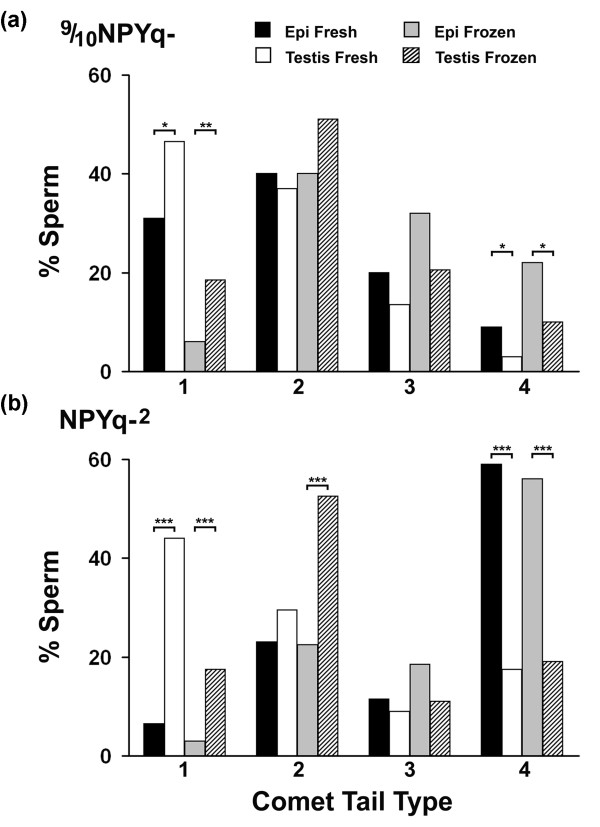
**The distribution of comet tail types in mice with severe NPYq deficiencies**. Four types of comet tail were differentiated: 1, short tail; 2, long tail, with majority of DNA still in the head; 3, long tail with DNA evenly distributed through out; 4, long tail with most of the DNA at the distal portion (baloon shape) [13]. The severity of DNA damage increases with tail type, from 1 to 4. Two males were used per genotype; four sperm groups (epididymal, frozen epididymal, testicular and frozen testicular) were examined per male; 100 sperm were scored per group per male. Statistical significance: *P < 0.05; **P < 0.01; ***P < 0.001 (Fisher's two-tailed exact probability test).

In addition to comet tail length, classification as to comet tail type can also give an indication of the level of DNA damage [13]. Based on the distribution of comet tail types, differences between epididymal and testicular sperm were observed in mice with severe NPYq deficiencies (Figure 3) but not in 2/3NPYq- mice. Thus, in NPYq-^2 ^and 9/10NPYq-, epididymal sperm yielded significantly fewer comets with tail type 1 (lowest damage) and significantly more comets with tail type 4 (most severe damage). The difference was more pronounced in NPYq-^2 ^than in 9/10NPYq-.

The comet data show that freezing increases DNA damage across all genotypes, that there is an increase in DNA damage relative to controls when the NPYq-deficiency exceeds that of 2/3NPYq-, and that epididymal sperm from mice with severe NPYq-deficiency are more susceptible to DNA damage than testicular sperm. We conclude that severe NPYq deficiency leads to DNA damage that is particularly marked in frozen epididymal sperm, and that this is likely to be the major factor underlying the very poor ICSI outcome using frozen epididymal sperm from NPYq-^2 ^males.

### NPYq deficiencies yield a high incidence of oocyte arrest and paternal chromosome breaks after ICSI

When collecting the ICSI activation data, we also collected data on the incidence of early post-fertilization oocyte arrest and of chromosome breakage in the paternal chromosome complements of zygotes, since both are known consequences of sperm DNA damage [13,14].

**Figure 4:**
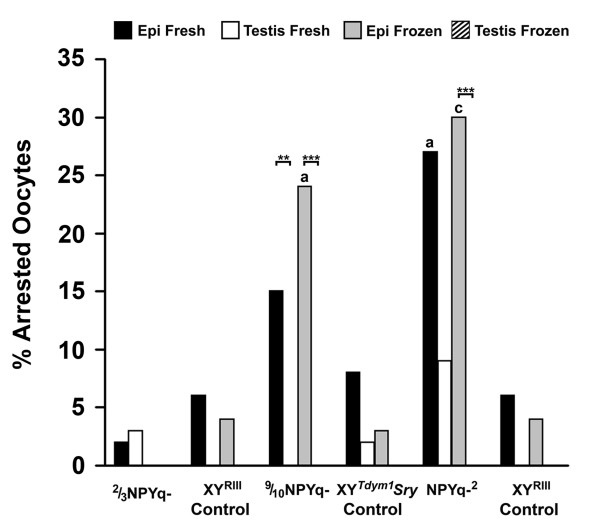
**Oocyte arrest after ICSI with sperm from mice with NPY deficiency**. Epididymal sperm from males with severe NPYq deficiency (9/10NPYq- and NPYq-^2^) but not males with moderate NPYq gene loss (2/3NPYq-) led to increased incidence of oocyte arrest compared to epididymal sperm from their appropriate controls, as revealed by Fisher's exact probability test. Statistical significance: a = *P *< 0.05; c = *P *< 0.001 versus matching sperm type in control. Epididymal sperm from males with severe NPYq deficiency (9/10NPYq- and NPYq-^2^) but not males with moderate NPYq gene loss (2/3NPYq-) led to increased incidence of oocyte arrest compared to testicular sperm, as revealed by Mantel-Haenszel chi square analysis. Statistical significance: **P < 0.01; ***P < 0.001. Two males were used per genotype; four sperm groups (epididymal, frozen epididymal, testicular and frozen testicular) were examined per male; approximately 20 (20.35 ± 6.94) oocytes were scored per group per male.

We compared the numbers of arrested and non-arrested oocytes between the NPYq-deficient genotypes and their controls using Fisher's exact test (Figure 4). This revealed increased oocyte arrest relative to controls for frozen epididymal sperm from 9/10NPYq- (*P *= 0.0172) and for fresh and frozen epididymal sperm from NPYq-^2 ^(*P *= 0.0347 and 0.0005, respectively). We then carried out 'within genotype' comparisons of epididymal and testicular sperm, both fresh and frozen, keeping the individual male data separate, using Mantel-Haenszel chi square analysis (Figure 4). The control genotypes did not show any significant differences in the incidence of oocyte arrest with epididymal as compared to testicular sperm, whether fresh or frozen. However, there was increased oocyte arrest with frozen epididymal sperm as compared to frozen testicular sperm from 9/10NPYq- and NPYq-^2 ^(*P *< 0.001 and *P *< 0.0005, respectively). There was also an increase in oocyte arrest with fresh epididymal as compared to fresh testicular sperm in 9/10NPYq- (*P *< 0.01). Thus, there is increased arrest when there is substantial NPYq deficiency.

**Figure 5:**
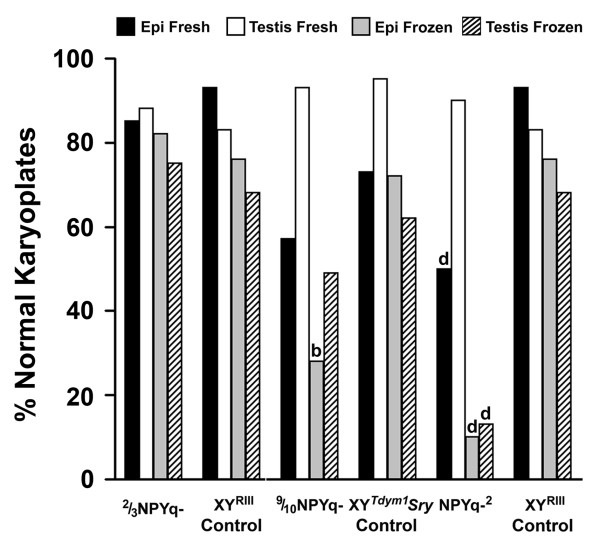
**Percentage of normal karyoplates after ICSI with sperm from mice with NPY deficiency**. Sperm from males with severe NPYq deficiency (9/10NPYq- and NPYq-^2^) but not males with moderate NPYq gene loss (2/3NPYq) led to increased incidence of abnormal karyoplates compared to respective sperm types from their appropriate controls, as revealed by Fisher's exact probability test. Statistical significance: b = *P *< 0.01; d = *P *< 0.0001 versus matching sperm type in control. Two males were used per genotype; four sperm groups (epididymal, frozen epididymal, testicular and frozen testicular) were examined per male; approximately 15 (15.88 ± 6.52) oocytes were scored per group per male.

For chromosomal breakage we first compared the number of oocytes with and without breaks in the paternal complement between the NPYq-deficient genotypes and their controls using Fisher's exact probability test (Figure 5). As with the incidence of oocyte arrest, the significant increases in the frequency of oocytes with chromosomal breakage were in 9/10NPYq- and NPYq-^2^. In 9/10NPYq- the effect was restricted to frozen epididymal sperm (*P *= 0.0059), whereas in NPYq-^2^, epididymal, frozen epididymal and frozen testicular sperm were affected (*P *= 0.0009, *P *< 0.0001 and *P *< 0.0001, respectively).

**Figure 6:**
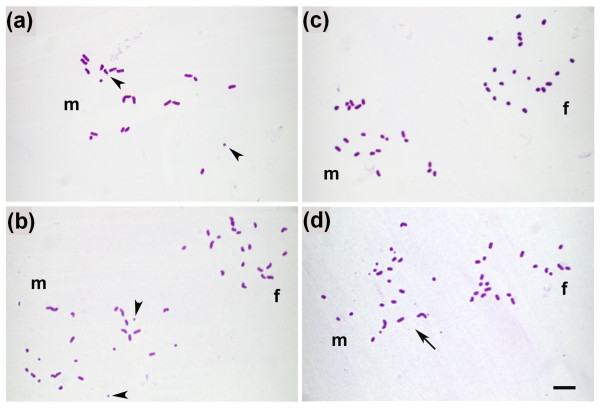
**Chromosome analysis after ICSI with sperm from mice with NPYq deficiencies**. **(a) **Fresh epididymal sperm from 9/10NPYq- male. **(b) **Fresh testicular sperm from 9/10NPYq- male. **(c) **Frozen testicular sperm from 9/10NPYq- male. **(d) **Frozen epididymal sperm from NPYq-^2 ^male. (a) Paternal chromosome complement (m) with 19 normal chromosomes and 3 fragments (examples shown with arrowheads). (b) Normal paternal (m) and maternal (f) chromosome complements each showing 20 chromosomes. (c) Normal maternal complement (f, n = 20) and paternal karyoplate with 18 normal chromosomes and 6 chromosome fragments (examples shown with arrowheads). (d) Mormal maternal chromosomes (f, n = 20) and paternal (m) complement with multiple chromosome aberrations (>10 fragments; arrow). Scale bar = 10 μm.

The paternal chromosome complements originating from NPYq-deficient mice had multiple chromosome and chromatid gaps, breaks and fragments, together with some abnormal chromosome configurations such as rings and exchanges (Figure 6). In order to better reflect the level of chromosome damage, we calculated the incidence of all chromosome aberrations for each sperm category for each male (aberration rate). The resulting data were analyzed by ANOVA.

**Figure 7:**
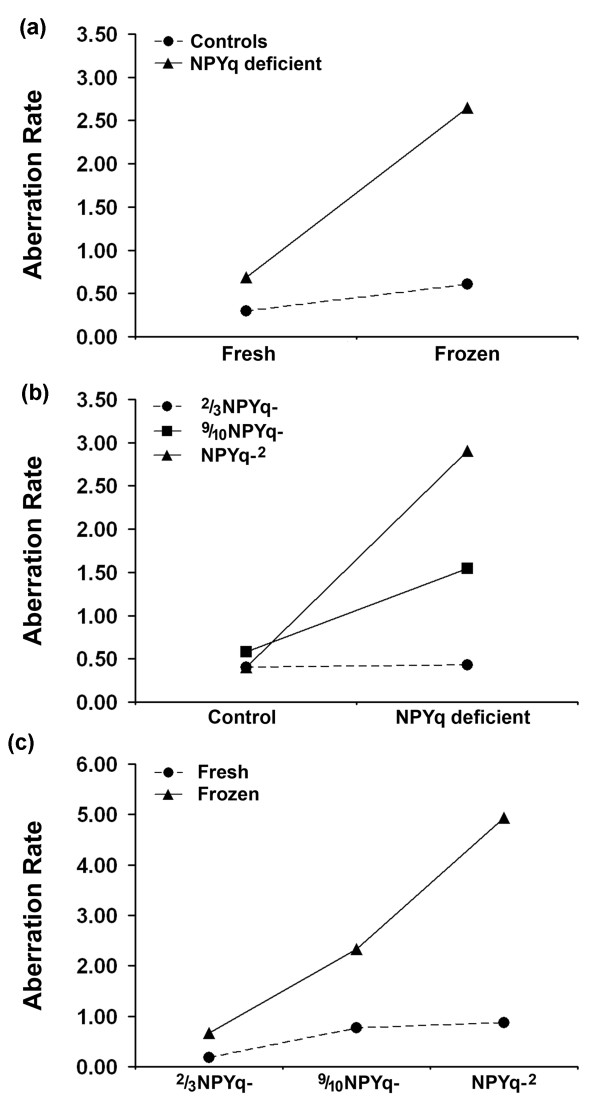
**Incidence of paternal chromosome breaks (aberration rate) in zygotes produced by ICSI with sperm from mice with NPYq deficiencies - ANOVA analysis**. **(a) **An increase in chromosome aberration rate due to sperm freezing in controls (*P *= 0.025) and NPYq deficient mice (*P *< 0.000001). **(b) **Comparison of NPYq-deficient genotypes with their respective controls showing the significant increase in chromosome aberration rate in 9/10NPYq- (*P *= 0.000145) and NPYq-^2 ^(*P *= 0.000019). (c) Genotype/sperm status interaction, showing that with increasing NPYq deficiency the increase in chromosome aberration rates is much more marked with frozen than fresh sperm (*P *= 0.000025). Two males were used per genotype; four sperm groups (epididymal, frozen epididymal, testicular and frozen testicular) were examined per male; approximately 15 (15.88 ± 6.52) oocytes were scored per group per male.

We first analyzed the data for the control males and this showed that sperm freezing increased the chromosome aberration rate (*P *= 0.025; Figure 7a) but there was a significant sperm source/status interaction (*P *= 0.027), with testicular sperm more sensitive to freezing than epididymal sperm. Comparison of each NPYq-deficient genotype with its matched control revealed that there was no increase in chromosome aberrations in 2/3NPYq-, but there was a 2.7-fold increase in 9/10NPYq- (*P *= 0.000145) and a 7.2-fold increase in NPYq-^2 ^(*P *= 0.000019) (Figure 7b). These markedly different aberration rates resulted in a highly significant effect of genotype (*P *= 0.000001) in the analysis of the three NPY-deficient genotypes in a single ANOVA, with aberration rate increasing with the extent of NPYq deficiency. However, this increase was predominantly seen with frozen sperm, resulting in a very significant genotype/sperm status interaction (*P *= 0.000025; Figure 7c) and a very significant affect of freezing overall (*P *< 0.000001; Figure 7a).

Based on the ANOVA analysis we conclude that in controls the freezing of testicular sperm leads to significant chromosome damage, and that severe NPYq deficiency leads to a marked increase in chromosome damage in response to sperm freezing, with testicular and epididymal sperm now being affected.

### Comparison of sperm comet and chromosome aberration data indicates that testicular sperm freezing impairs sperm DNA damage repair in the oocyte

There is substantial evidence showing that oocytes have DNA repair machinery present at fertilization that enables DNA damage in the sperm nucleus to be repaired [15]. The chromosome aberrations present in fertilized oocytes are therefore a manifestation of prior DNA damage that cannot be repaired by the oocyte. For six of the males in the present study the same sperm samples were used for the sperm comet and oocyte paternal chromosome complement analyses, so a direct comparison of these sets of data should highlight those factors that lead to irreparable DNA damage.

The six males for which both sets of data are available are XY^RIII ^(n = 1), 2/3NPYq- (n = 2), 9/10NPYq- (n = 1) and NPYq-^2 ^(n = 2). Because 2/3NPYq- males (with moderate NPYq deficiency) do not manifest any significant differences from XY^RIII ^in either assay, we treated the first three males as one group (group 1, G1). The 9/10NPYq- and NPYq-^2 ^males (severe NPYq deficiency) constituted the second group (group 2, G2), which differed markedly from their controls in both assays. The two groups were first compared by ANOVA. For sperm comet tail length there was a 38% increase as a consequence of severe NPYq deficiency (*P *< 0.000001); epididymal sperm were preferentially affected in G2 whereas in G1 it was the testicular sperm that had the longer sperm comet tails (group/sperm source interaction, *P *= 0.0032). For chromosome aberration rate there was an almost six-fold increase as a consequence of the NPYq deficiency (*P *= 0.000013); sperm from G2 males were much more sensitive to freezing than those from G1, resulting in a significant group/sperm status interaction (*P *= 0.00070).

**Figure 8:**
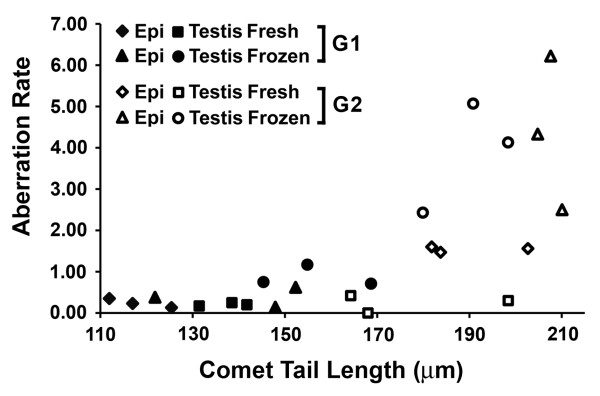
**Comparison of comet assay and chromosome aberration analysis - ANOVA analysis**. Comet tail length versus aberration rate scatter plot with distinction between sperm source and sperm status. Group 1 (G1, n = 3) = controls + 2/3NPYq-; group 2 (G2, n = 3) = 9/10NPYq- and NPYq-^2^.

Within group comparisons established that in G1 males there was more DNA damage (comet assay) in testicular sperm than epididymal sperm (*P *= 0.0014) and for both sperm sources the level of damage was markedly increased by freezing (*P *= 0.00096), but the chromosome aberration rates were only markedly elevated with frozen testicular sperm (*P *= 0.031 for source/status interaction), indicating that most of the damage due to freezing in epididymal sperm was repaired in the oocyte. However, in G2 males, the increase in sperm DNA damage due to freezing was reflected in markedly increased chromosome aberration rates with both sperm sources (*P *= 0.001). In addition, there was a significant increase in chromosome aberrations with fresh epididymal sperm as compared to fresh testicular sperm (*P *= 0.000568). These differing effects of sperm source and sperm freezing between the two groups became apparent when the comet tail length and chromosome aberration rate data were plotted as a scatter plot (Figure 8). In summary: frozen testicular sperm from controls and 2/3NPYq- (G1) have DNA damage that is not resolved in the oocyte, with a consequent increase in chromosome aberrations; and in males with severe NPYq-deficiency (G2), there is a marked increase in sperm DNA damage in testicular and epididymal sperm. Similarly to G1, frozen testicular sperm have some DNA damage that is not resolved in the oocyte, but in contrast to G1 this is also true of fresh and frozen epididymal sperm.

### Sperm from males with NPYq deficiencies have impaired membrane integrity and abnormal chromatin condensation as shown by electron microscopy analysis

Transmission electron microscopy was used to determine membrane integrity and appearance of chromatin in epididymal sperm from mice with NPYq deficiencies. Three males per genotype were examined (except for 9/10NPYq-, for which only two males were tested) and 100 sperm heads were scored per male.

**Figure 9:**
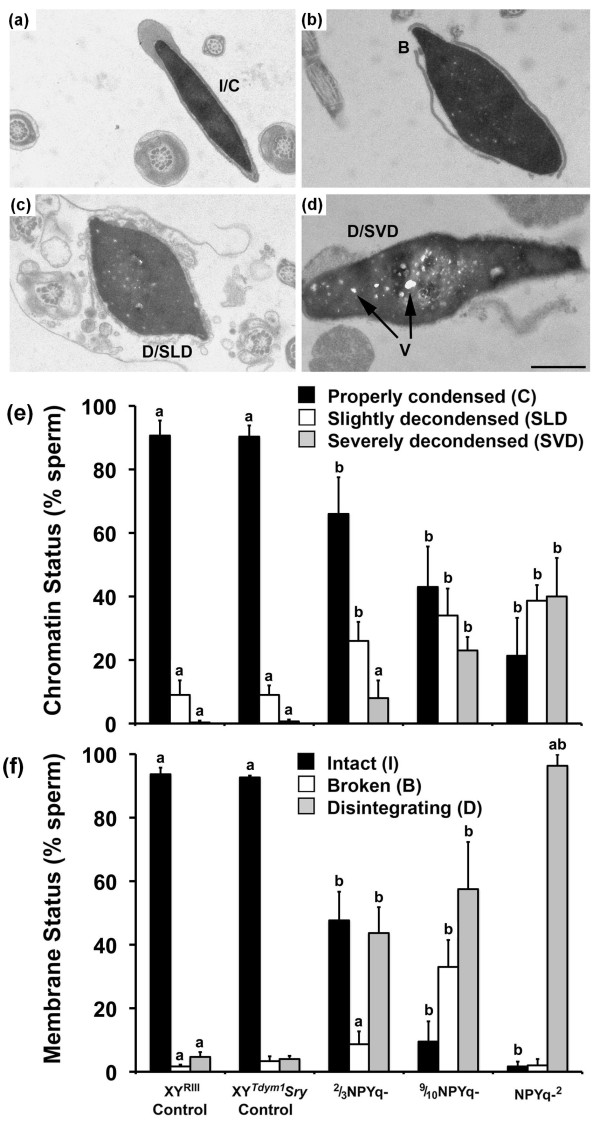
**Transmission electron microscopy analysis of sperm from mice with NPYq deficiencies**. **(a-d) **Examples of sperm with various membrane and chromatin condensation deficiencies. **(e, f) **Analysis of frequency of sperm with various chromatin and membrane integrity deficiencies. When examining chromatin condensation we observed the presence of bright white spots (voids; V), which appeared always in conjunction with severely decondensed chromatin and were present exclusively in sperm from mice with NPYq deficiencies. Scale bar = 1 μm. Three males per genotype were examined (except for 9/10NPYq-, for which only two males were tested); 100 sperm heads were scored per male. Each bar represents mean ± standard deviation. Statistical significance: a = different from other categories within genotype; b = different from respective category in control (Fisher's exact probability test).

With respect to membrane integrity, we assigned examined sperm heads into three categories reflecting progressive membrane damage: I, intact; B, broken; and D, disintegrating (Figure 9). There were no differences when the incidences of specific categories were compared across control genotypes; almost all sperm had intact membranes (approximately 93%). NPYq-^2 ^mice had predominantly sperm with a disintegrating membrane (approximately 96%) and 9/10NPYq- had the majority of sperm with either broken or disintegrating membrane (>90%). In 2/3NPYq-, most sperm had either intact (approximately 48%) or disintegrating membranes (approximately 44%). All NPYq-deficient genotypes had significantly fewer sperm with intact membrane and significantly more sperm with disintegrating membrane than their respective controls. When NPYq-deficient genotypes were compared against each other, the ranking reflecting the severity of membrane integrity impairment was: NPYq-^2 ^> 9/10NPYq- > 2/3NPYq-.

When examining chromatin condensation we categorized sperm into three categories: those with properly condensed (C), slightly decondensed (SLD) and severely decondensed (SVD) chromatin (Figure 9). All controls had the vast majority of sperm with properly condensed chromatin (>90%). In 2/3NPYq- males, sperm with properly condensed chromatin predominated (66%) and less than 10% had severely decondensed chromatin. In 9/10NPYq- and NPYq-^2 ^males more than half of sperm had decondensed chromatin (approximately 57% and approximately 79%, respectively). All NPYq-deficient genotypes had fewer sperm with properly condensed chromatin and more sperm with slightly and severely decondensed sperm than their respective controls.

When membrane integrity and chromatin condensation status were compared, the test for linear trend in proportions [16] confirmed a significant correlation between the maintenance of sperm membrane integrity and proper chromatin condensation (*P *< 0.001).

Overall, the data show that mice with NPYq deficiencies exhibit membrane damage and abnormal chromatin condensation in sperm, which increases in parallel with the level of NPYq gene loss.

### Sperm from males with NPYq deficiencies have impaired protamine processing

**Figure 10:**
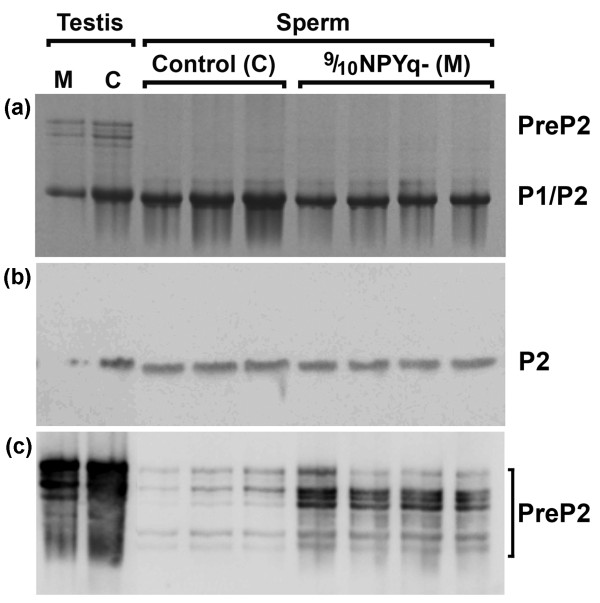
**Sperm nuclear protein analysis**. A representative acid-urea gel separation of nuclear proteins extracted from epididymal sperm and testes of 9/10NPYq- mutant (M, n = 4) and XY^Tdym1^Sry control (C, n = 3) mice. **(a) **Coomassie blue stained gel. **(b) **Immunoblot with Hub2B antibody recognizing P2. **(c) **Immunoblot with preP2 antibody recognizing preP2. P1 and P2, protamine 1 and 2, respectively; PreP2, premature forms of protamine 2.

To test whether increased sperm DNA damage resulted from abnormal protamination of sperm chromatin, we examined epididymal sperm from mice with NPYq deficiencies for the presence of premature protamine forms, with testis samples providing positive controls. Sperm nuclear protein samples corresponding to the same sperm number were separated on acid-urea polyacrylamide gels. At least two gels were run and at least three males were tested per genotype (Table 2). No premature protamine P2 bands were detected on Coomassie blue stained gels, in any of the tested genotypes (Figure 10a). However, when the gels were blotted with preP2 antibody, which recognizes premature protamine 2 forms, bands were detected in samples from 9/10NPYq- mice and their controls on two of the four gels (Figure 10c, Table 2). The intensity of preP2 bands was significantly higher for 9/10NPYq- mice than for controls (*P *= 0.0001). No preP2 was detected in samples from 2/3NPYq and NPYq-^2 ^mice, in the latter genotype perhaps due to the lowest number of mice tested. Additional evidence of abnormal protamination came from measuring band intensities for mature protamines. On Coomassie blue stained gels, there was no reduction in band intensity relative to controls in 2/3NPYq-, but in 9/10NPYq- the band intensity was reduced by 37% (*P *= 0.002) and in NPYq-^2 ^males band intensity was reduced by 71%, although this reduction was not statistically significant with the limited number of samples analyzed (Table 2). When the membranes were blotted with Hub2B antibody recognizing mature protamine 2, the same pattern of decreasing levels of the mature form with increasing NPYq deficiency was observed, there being no reduction in 2/3NPYq-, a 12% reduction in 9/10NPYq- (*P *= 0.00005) and a 44% reduction in NPYq-^2 ^males (not significant) (Figure 10b, Table 2).

**Table 2:** Western blot analysis of protamines in mice with NPYq deficiencies

	Mean band intensity^b ^(±SEM) representing mature protamine forms			
			
Mice examined^a^	Coomassie	Hub2B (protamine 2)	Mice examined^a^	Mean band intensity^b ^(±SEM) representing immature protamine 2 (PreP2)
2/3NPYq- (n = 7)	141.9 ± 4.6	125.3 ± 4.6	-	-
XY^RIII ^(n = 6)	138.2 ± 5.0	124.9 ± 5.0		
				
9/10NPYq- (n = 7)	82.5 ± 4.8*	43.5 ± 0.3***	9/10NPYq- (n = 5)	89.2 ± 4.5**
XY^*Tdym1*^*Sry *(n = 6)	131.0 ± 5.2	49.3 ± 0.3	XY^*Tdym1*^*Sry *(n = 4)	53.8 ± 5.0
				
NPYq-^2 ^(n = 3)	22.1 ± 16.2	20.8 ± 6.6		
XY^RIII ^(n = 3)	76.0 ± 16.2	36.9 ± 6.6	-	-

^a^At least two separate gels were run for each NPYq deficient genotype (2/3NPY-, two gels; 9/10NPYq-, four gels; NPYq-^2^, two gels). For PreP2, data were obtained from only two gels. ^b^The data were analyzed by two-way ANOVA with gel and genotype as factors, from which the genotype means and errors are derived. Statistical significance: *P < 0.005; **P < 0.0005; ***P < 0.00005. The lack of statistical significance for the reduction observed in NPYq-^2 ^was almost certainly due to the limited number of samples. SEM, standard error of the mean.

In summary, the data indicate that mice with severe NPYq deficiencies have impaired sperm chromatin protamination leading to an increase in premature protamine forms and a decrease in mature protamine forms in epididymal sperm.

## Discussion

The aim of the present study was to provide an explanation for the very poor ICSI success when using frozen epididymal sperm from mice with severe NPYq deficiency. The results show that the major underlying cause is an increase in DNA damage that particularly affects epididymal sperm, and that is further increased by sperm freezing. This DNA damage included damage that was not reparable by the DNA repair machinery present in the oocyte, resulting in a marked increase in chromosome aberrations in the paternal chromosome complement following ICSI.

What is the origin of the increased sperm DNA damage in severely NPYq-deficient mice that is already detected in testicular sperm and has increased markedly by the time the sperm have reached the cauda epididymis? The origin of sperm DNA damage is a subject of substantial research and much debate. Aitken *et al*. [17,18] have proposed a two step model in which sperm exiting the testis with chromatin remodeling defects, such as incomplete protamination, histone retention and poor compaction, subsequently acquire membrane and DNA damage as they transit the epididymis. Several proposed, not mutually exclusive causes of this DNA damage include direct damage by reactive oxygen species [17,19] and the action of endonucleases present either within the sperm or in epididymal fluid [13,20-22]. However, there is also evidence that damaged spermatozoa can be selectively removed during epididymal transit [23,24], so the level of DNA damage detected in sperm from the cauda epididymis depends on the balance between the generation of DNA damage and the removal of sperm with DNA damage during epididymal transit. Thiol-cross linking, which takes place during epididymal maturation [25-27], increases overall chromatin compaction. If sperm exiting testes already have some chromatin defect, epididymal maturation may be impaired or may not take place.

In the case of severely NPYq-deficient mice, the primary defect must arise during spermiogenesis since this is when the NPYq-encoded genes are expressed in normal mice [3,5,28]. In keeping with the two step model for the generation of DNA damage in epididymal sperm [18], we have found that epididymal sperm from NPYq deficient mice have defective protamination and chromatin compaction, with associated membrane damage. However, testicular sperm from these mice already have elevated levels of DNA double-strand breaks (DSBs) as detected by the comet assay. Transient DSBs are generated by topoisomerase TOP2B during normal sperm chromatin remodeling and it has been suggested that inefficient repair of these breaks may occur in the context of disturbed protamination [29,30].

The exact mechanistic link between NPYq deficiency and the chromatin changes is difficult to determine because of the major transcriptional changes in spermatids that are associated with NPYq deletions. More than 200 genes were found to be differentially expressed in mice with NPYq deficiencies, as compared to wild-type controls, the most striking change being the over-expression of sex chromosome linked genes; among the misregulated genes are some that are implicated in chromatin remodeling during spermiogenesis (NPYp-linked *H2al2y*, X-linked *H2al1 *and *Cypt*, and autosomal *Hist1h3*, *Hist1h4*, *Chaf1b *and *Speer*) [28,31]. Given the extent of this transcriptional regulation, it is perhaps unsurprising that there are also changes in the pattern of spermatid chromatin modifications [32]. The majority of these genes are also misregulated in mice with a specific small interfering RNA-mediated disruption of the function of the multi-copy NPYq gene *Sly*. These mice are near sterile and recapitulate most of the features associated with severe NPYq deficiency, including altered patterns of spermatid chromatin modifications, thus demonstrating that this multi-copy NPYq gene plays a key role in regulating spermiogenic gene expression [31]. No homologues of *Sly *or the other multi-copy mouse NPYq genes have been identified on the human Y chromosome. Nevertheless, multi-copy testis expressed genes are a feature of the human Y [33,34] and the multi-copy testis specific gene *RBMY *has a multi-copy homologue (*Rbmy*) on the mouse Y short arm [35]. Thus, the two species have in common the fact that genes with a presumed or demonstrated spermatogenic role are often amplified on the Y.

DSBs are extremely hazardous lesions that must be repaired before cell division occurs if cell death is to be avoided. It is well established that oocytes have the DNA damage repair machinery in place to repair DSBs once the sperm chromatin begins to decondense as it is deprotaminated [15,36-39]; this initial repair utilizes non-homologous end joining (NHEJ) to re-ligate the broken ends [15] and our data suggest this mechanism is sufficient to deal with any DSBs in epididymal sperm from controls, even when the numbers are increased by freezing. However, with fresh epididymal sperm from NPYq-deficient mice there is an elevation in the chromosome aberration rate manifested as broken chromosomes and chromosome rearrangements; with freezing this is further increased, leading to elevated levels of chromosome aberrations also in testicular sperm from control and NPYq-deficient males. These chromosome aberrations are manifestations of unrepaired DSBs. Chromosome rearrangements occur when the 'wrong' DNA ends are ligated, and constitute evidence that the original broken ends have not been maintained in juxtaposition; instead there has been a chance juxtaposition of non-matching ends that are then ligated by NHEJ. The chromosome breaks almost certainly represent the separation of broken DNA ends without the chance finding of an alternative partner. How broken DNA ends might be held in juxtaposition in the protaminated chromatin of the sperm head is not known; nevertheless, the data we have obtained suggest that with incompletely protaminated DNA, as in testicular sperm and in epididymal sperm from NPYq deficient mice, the nuclear matrix/protein scaffold holding the DNA ends together is susceptible to disruption by freezing.

What are the broader implications of the present findings? ICSI is widely used to treat human male infertility due to oligozoospermia or teratozoospermia that is usually associated with sperm defects, such as incomplete protamination and incomplete compaction of the chromatin [40] (reviewed in [41,42]). Thus, the severely NPYq-deficient mice studied here are a useful mouse model for assessing the consequences of these defects for the offspring. It is also important to consider the implications of the additional DNA damage that arises as a consequence of freezing in the NPYq-deficient mice as well as in the controls. With freezing and NPYq deficiency, almost none of the embryos transferred reached term; this is unsurprising given the level of chromosome breaks and rearrangements generated, which would be expected to cause early embryonic lethality. This raises a number of issues as to how to maximize ICSI success and how to minimize mutational load in the offspring. In the context of ICSI success it seems clear that in situations where there is increasing DNA damage during epididymal transit, as is the case with NPYq deficiency, it is better to use testicular sperm; however, freezing should be avoided. Improved ICSI success with testicular as compared to epididymal sperm has previously been reported for mice heterozygous for two different but semi-identical translocations [43] and in mice deficient for transition proteins [11,44], as well as in infertile men with sperm DNA damage [45]. A caveat is that in the present study sperm freezing was done without cryoprotectant, but with the method developed to keep DNA damage to a level comparable to freezing with cryprotectant [46,47]. Indeed, substantial freezing-induced DNA damage has been reported even with mouse sperm frozen with cryoprotectant [48] and with cryopreserved human sperm [49-51].

As to mutational load, NHEJ is inevitably mutagenic even when the correct partners are re-ligated [52], so it is expected that the mutational load will increase as the DNA damage increases; this is supported by a recent study of offspring generated by ICSI using frozen-thawed mouse sperm [53]. It has also been argued that disturbances of chromatin remodeling can generate 'epi-mutations' that can contribute to the mutational load across generations [54]. Aside from the implications for treating human infertility by ICSI, our findings raise questions as to the mutational load associated with the use of cryopreserved sperm in general. With the extensive single nucleotide polymorphism mapping panels now available that provide sequence-based, genome-wide markers, it should be possible to screen for DNA sequence changes arising as a consequence of sperm freezing.

## Conclusions

We provide the first evidence on sperm DNA damage in conjunction with deletions of the Y chromosome long arm (NPYq) in mice, with support for the underlying mechanism. NPYq-deficient mice serve as a model for human infertility cases due to Y chromosome deletions and/or cases associated with sperm DNA damage and abnormal sperm chromatin compaction. In addition to demonstrating the involvement of NPYq-encoded genes in regulating chromatin remodeling during spermiogenesis, the study also provides important insights into the regulation of sperm DNA integrity after they are released from the testis, in the epididymis and in the oocyte after fertilization. In the context of other recently published work, our study points to there being an increased mutational load across generations as a consequence of assisted reproduction with sperm resulting from defective chromatin compaction during spermiogenesis or sperm subjected to cryopreservation.

## Materials and methods

### Chemicals

Mineral oil was purchased from Squibb and Sons (Princeton, NJ, USA); pregnant mares' serum gonadotrophin (eCG) and human chorionic gonadotrophin (hCG) from Calbiochem (San Diego, CA, USA). All other chemicals were obtained from Sigma Chemical Co. (St Louis, MO, USA) unless otherwise stated.

### Animals

Six- to twelve-week-old B6D2F1 (C57BL/6J × DBA/2) females (NCI, Raleigh, NC, USA) were used as oocyte donors for ICSI. The mice of interest in this study were three mutant mice with progressive NPYq deficiency: XY^RIII^qdel (subsequently called 2/3NPYq) - these males have a RIII strain-derived Y chromosome with a deletion removing approximately two-thirds of NPYq; XY^*Tdym1*^qdel*Sry *(subsequently called 9/10NPYq-) - these males have a 129 strain-derived Y chromosome with an 11-kb deletion removing the testis determinant *Sry *[55] that is complemented by an autosomally located *Sry *transgene [35], together with a deletion removing approximately nine-tenths of NPYq [7]; and XY*^X^*Sxr*^a ^(subsequently called NPYq-^2^) [8] - in these males the only Y specific material is provided by the Y^RIII ^short arm derived sex reversal factor *Sxr*^a ^that is attached distal to the PAR of the Y*^X ^chromosome. The Y*^X ^chromosome is an X chromosome with a very large deletion removing most of the X-specific region but leaving an intact PAR and X PAR boundary, together with the X centromere [56]. NPYq-^2 ^males lack the entire Y-specific (non-PAR) gene content of Yq in addition to a reduction in copies of *Rbmy *on Yp. The control for 2/3NPYq- and NPYq-^2 ^mice is XY^RIII^, and for 9/10NPYq- is XY^*Tdym1*^*Sry *mice, which carry the same Y chromosome on which the deletion variant arose. All mice were produced 'in-house' by either breeding or assisted reproduction, and were on a predominantly C57BL/6 genetic background (more than six generation backcross from MF1 for all males except for NPYq-^2^, which were either 62.5% or 81.25% C57BL/6). The mice were maintained in accordance with the NCR 'Guide for Care and Use of Laboratory Animals' in rooms at 22°C with 14 h light/10 h dark, and fed *ad libitum*.

### Gamete collection and embryo culture

Oocyte collection and subsequent oocyte manipulation, including microinjections, were done in HEPES-buffered CZB medium (HEPES-CZB) [57], with subsequent culture in CZB with an atmosphere of 5% CO_2 _in air [58]. To obtain testicular sperm a portion of testis was cut off and minced in ETBS (an EGTA Tris-HCl-buffered solution consisting of 50 mM EGTA, 50 mM NaCl, and 10 mM Tris-HCl buffer, pH 8.2-8.5 [46]) or HEPES-CZB to release spermatogenic cells. To obtain epididymal sperm the contents of the caudae epididymides were expressed with needles and placed in HEPES-CZB, ETBS or phosphate-buffered saline. Spermatozoa were allowed to disperse for 2 to 3 minutes at room temperature. The samples of testicular or epididymal cell suspension were used for ICSI, comet assay, preparation for transmission electron microscope (TEM) analysis or sperm nuclear protein isolation immediately after dispersion, or were subjected to freezing. In some cases epididymal and testicular samples were sonicated before freezing and/or comet assay (65 output, 10 pulses 1 s each). After sonication the cell suspension was layered over a two-layer (1.8 to 2.2 M) sucrose gradient and centrifuged (400 *g*, 20 minutes). The pellet was resuspended in HEPES-CZB or ETBS, and checked under the light microscope to confirm that only sonication-resistant cells (sperm and elongated spermatids) were present.

### Epididymal and testicular sperm freezing

Aliquots of 10 μl epididymal sperm or testicular cell suspension in ETBS were loaded in 0.25 ml straws (Edwards Innovations, Spring Valley, VA, USA). Each straw was sealed with Critoseal (Oxford Labware, St Louis, MO, USA) and placed in a plastic holder floating on the surface of the liquid nitrogen for 10 minutes before immersion. For thawing, the straws were removed from the storage container and immersed in a water bath at 37°C for 10 minutes and the contents expressed into a Petri dish. Spermatozoa were used immediately for ICSI or other analyses.

### Intracytoplasmic sperm injection

ICSI was carried out as previously described [59] within 1 to 2 h from oocyte collection and with live sperm randomly chosen for the injections. Sperm-injected oocytes were transferred into CZB medium and cultured at 37°C. The survival of ICSI oocytes was scored 1 to 2 h after the commencement of culture. The activation of ICSI oocytes was scored 6 h after the commencement of culture; the oocytes with two well-developed pronuclei and extruded second polar body were considered activated. The number of two-cell embryos ('fertilized') was recorded after 24 h in culture.

### Chromosome analysis

Chromosome preparation and analysis were performed as previously described [13,14]. The Y chromosome of 9/10NPYq- males and the Y*^X ^chromosome of NPYq-^2 ^males are minute and the latter males also generate some sperm lacking a sex chromosome [7,8]. Therefore, for these two genotypes the presence of one small variant and/or lack of one chromosome in the paternal chromosome complement were considered normal. For control males the chromosomes of a spermatozoon were considered normal when an oocyte contained 40 normal metaphase chromosomes, 20 maternal and 20 paternal. It was not always possible to distinguish between chromosomes of paternal and maternal origin. However, since oocyte chromosomes rarely show structural aberrations at first cleavage metaphase after parthenogenetic activation [14], any abnormal chromosomes within fertilized oocytes were considered to be of sperm origin. Among the chromosome aberrations, we differentiated between minor (1 to 9 aberrations per karyoplates examined) and multiple (>9, scored always as 10 aberrations per oocyte). In addition to scoring normal versus abnormal karyoplates, we also calculated the incidence of chromosome aberrations, that is, aberration rate, which represents the total number of aberrations divided by the number of oocytes examined.

### Comet assay

Sperm DNA fragmentation was assessed using a Trevigen Comet Assay kit (Trevigen, Gaithersburg, MD, USA, catalog no. 4250-050-K) under neutral conditions as previously described [13]. One-hundred DNA tails were photographed and analyzed per slide and two males were analyzed per genotype. The length of each tail was measured from the center of the comet head to the end of the tail by Image J software [60] and classified into one of the four categories [13].

### Transmission electron microscope analysis

Epididymal sperm were fixed in 2.5% glutaraldehyde, 0.1 M sodium cacodylate, 2 mM calcium chloride, pH 7.4, 1 h, then postfixed with osmium tetroxide (1% in 0.1 M cacodylate buffer, 1 h), dehydrated in ethanol, substituted with propylene oxide, and embedded in LX-112 epoxy resin. Ultra-thin sections (60 to 80 nm) were collected on Formvar-coated copper grids, double stained with uranyl acetate and lead citrate, and viewed with a LEO 912 (Zeiss) TEM, and photographed at 8,000× original magnification.

### Preparation and analysis of sperm nuclear proteins by immunoblot

All extraction and preparation procedures were performed as described previously [61] except that sperm tails were removed by chemical treatment rather than sucrose centrifugation [62]. The proteins were separated by electrophoresis in acid-urea 15% or 20% polyacrylamide gels and either stained with Coomassie blue or used for immunoblotting; an aliquot of sperm proteins corresponding to 4.5 × 10^6 ^cells was loaded per each lane. Antibody detection was performed as described previously [61]. Briefly, the nuclear proteins were transferred to a nitrocellulose membrane in 7% acetic acid using Criterion wet blotting unit (BIORAD, Los Angeles, CA, USA). After blocking, the membrane was incubated for 2 h at room temperature with preP2 antibody recognizing the precursor domain of protamine 2 [12] (1:9,000; courtesy of Marvin Meistrich, University of Texas) and/or antibody Hup2B detecting both protamine 2 [63] (1:500,000; courtesy of Rod Balhorn, Lawrence Livermore Laboratories). Incubation with the corresponding secondary antibody conjugated with horseradish peroxidase and detection by chemiluminescence were carried out as described by the manufacturer (Amersham Pharmacia, Piscataway, NJ, USA). Band intensities (from Coomassie blue staining or western blot detection) were quantified using Photoshop software.

### Statistics

In the analysis of oocyte activation, oocyte arrest, and incidence of abnormal karyoplates, Mantel-Haenszel chi square test was used for 'within genotype' comparisons and Fisher's exact test was used to compare NPYq-deficient genotypes with their respective controls, and also to analyze the incidence of comet tail types and the TEM data. ANOVA (Generalized Linear Model as provided by NCSS Statistical Analysis Software (Kaysville, UT, USA)) with genotype, sperm source (epididymis or testis) and sperm status (fresh or frozen) as factors was used for the analysis of the oocyte activation data (after transforming percentage data to angles), comet tail lengths and for chromosome aberration rates, and for the analysis of western blot band intensities with gel and genotype as factors.

## Abbreviations

DSB: double-strand break; NHEJ: non-homologous end joining; 2/3NPYq: mice with a deletion removing approximately two-thirds of NPYq; 9/10NPYq: mice with a deletion removing approximately nine-tenths of NPYq; ICSIL intracytoplasmic sperm injection; NPYq, non-PAR: Y chromosome long arm, male specific Y chromosome long arm; NPYq-^2^: mice lacking the entire NPYq.

## Authors' contributions

MAW conceived, designed and coordinated the study. YY, JMR and ZS performed experiments: YY microinjection and chromosome analysis; JMR genotyping, comet assay and TEM; ZS protamine assays. MAW and PSB carried out data processing, statistical analyses, and prepared the manuscript. All authors read and approved the final manuscript.
